# Effect of Varying Amine Functionalities on CO_2_ Capture of Carboxylated Graphene Oxide-Based Cryogels

**DOI:** 10.3390/nano10081446

**Published:** 2020-07-24

**Authors:** Alina I. Pruna, Arturo Barjola, Alfonso C. Cárcel, Beatriz Alonso, Enrique Giménez

**Affiliations:** 1 Instituto de Tecnología de Materiales, Universitat Politècnica de València (UPV), Camino de Vera s/n, 46022 Valencia, Spain; apruna@itm.upv.es (A.I.P.); arbarrui@doctor.upv.es (A.B.); acarcel@upv.es (A.C.C.); 2 Graphenea S.A., Paseo Mikeletegi 83, 20009 San Sebastián, Spain; b.alonso@graphenea.com

**Keywords:** graphene oxide, amine, cryogel, CO_2_ capture

## Abstract

Graphene cryogels synthesis is reported by amine modification of carboxylated graphene oxide via aqueous carbodiimide chemistry. The effect of the amine type on the formation of the cryogels and their properties is presented. In this respect, ethylenediamine (EDA), diethylenetriamine (DETA), triethylenetetramine (TETA), were selected. The obtained cryogels were characterized by Fourier Transformed Infrared spectroscopy, thermogravimetric analysis, X-ray spectroscopy, and Scanning electron microscopy. The CO_2_ adsorption performance was evaluated as a function of amine modification. The results showed the best CO_2_ adsorption performance was exhibited by ethylenediamine modified aerogel, reaching 2 mmol g^−1^ at 1 bar and 298 K. While the total N content of the cryogels increased with increasing amine groups, the nitrogen configuration and contributions were determined to have more important influence on the adsorption properties. It is also revealed that the residual oxygen functionalities in the obtained cryogels represent another paramount factor to take into account for improving the CO_2_ capture properties of amine-modified graphene oxide (GO)-based cryogels.

## 1. Introduction

The developments in nanotechnology and innovations in graphene research indicated graphene cryogels as a novel class of three-dimensional (3D) architecture with prodigious potential in varying applications including CO_2_ capture, energy storage or pollutant adsorption. The myriad of applications arise from the outstanding properties of these cryogels such as their low density, high specific area, mechanical strength and electrical conductivity [1,2,3,4].

The most effective approach to integrate graphene into such bulk materials is the self-assembly of graphene oxide (GO) sheets [5,6,7]. In this respect, one of the most used and preferred methods is the hydrothermal one, due to its low cost and easy implementation. By applying the simultaneous reduction and self-assembly of GO in the presence of amines and a subsequent freeze-drying procedure, aerogels could be synthesized to tackle the urgent environmental matter of highly selective and efficient CO_2_ capture [8,9,10,11,12,13,14].

The practical implementation of amine bulk sorbent materials generally requires a high surface area, high pore volume, high amine content, amine stability, etc. [15,16,17]. However, there are large discrepancies in the most affecting parameters towards tailoring the CO_2_ capture properties. For example, while the specific surface area of a polyethyleneimine-modified GO was reportedly low, its CO_2_ adsorption capacity reached values as high as 1.9 mmol g^−1^ at 298 K and 1 bar [18].

The approaches to improve the structure and adsorption properties of these macroscopic materials mainly refer to adjusting the properties of GO and to the control of the functionalization degree. Concerning GO, approaches such as narrowing of GO size distribution [19] by employing different mesh size of the parent graphite for oxidation or the control of the oxidation degree were studied [20]. Our group has previously reported on the effect of simple hydrothermal synthesis conditions on the gelation and formation, as well as the effect of GO synthesis conditions on the CO_2_ capture properties of ethylenediamine (EDA)-impregnated GO-based 3D monoliths [8,9]. Other studies considered the impregnation with amines such as EDA for obtaining 3D monoliths with improved conductivity and mechanical properties [21].

Concerning the functionalization of GO, the incorporation of nitrogen atoms is commonly applied in order to generate various active sites. The nitrogen content was reported to be greatly influenced by the choice of solvent [16,17], which is usually an organic one. Various reports indicated the N configuration has marked effect on CO_2_ conversion and uptake, selectivity and activity of doped material [8,9,22,23,24,25,26,27]. The type of N configuration could be tailored by suitable synthesis conditions. On the other hand, the residual oxygen functional groups in the graphene aerogels were shown to participate, as well, to the interaction with CO_2_, influencing their adsorption capacity and selectivity [28,29].

The preferred approaches to modify the GO surface with amine molecules include impregnation or covalent functionalization [30]. Despite its simplicity, time and cost efficiency, the physical immobilization of amines achieved by the simple wet impregnation route mostly rely on weak interactions between the amine and GO which could compromise the stability and lifetime of the sorbent. This approach could also affect the level of amine loading, which is known to be directly linked to the CO_2_ adsorption capacity [31]. As amine leaching is an aspect that needs to be considered for long-term viability as a CO_2_ capture agent [19,32,33], the synthesis conditions and operating ones, such as high temperatures must be carefully selected. A proposed approach to improve the loading and amine stability is to exploit the reactivity of the oxygen groups on GO to covalently functionalize the GO surface, similarly to other sorbents [20,34,35,36].

Therefore, control of the GO functionalization results essential for the design of aerogels with improved CO_2_ adsorption properties. It should be noted that the different protocols employed for GO preparation were reported to induce inhomogeneity between the studied samples. As the GO is decorated with different oxygen groups, varying simultaneous derivatization reactions, including epoxy ring opening and the amidation of carboxylic acids of GO, may be induced [15] and, as such, the efficiency evaluation of such reactions is a difficult task. Under these aspects, the carboxylation of GO was suggested as a suitable approach to obtain a precursor material with a similar oxidation degree for the functionalization [37]. Moreover, the amine grafting ability of the oxygen groups on GO was shown to be higher for carboxyl ones [38].

On the other hand, a high nitrogen content and desired nitrogen configuration could be achieved by adjusting the chain length and the number of functionalities in the amine employed for functionalization [16]. These parameters were shown to affect the CO_2_ uptake performance also in terms of suitable space between the functionalized GO layers for the reaction with the gas [39]. For example, EDA resulted in better uptake reaching 1.46 mmol g^−1^ than its counterparts functionalized with butanediamine and hexanediamine [40]. The type of amine is another important aspect as well, with the secondary types being indicated as a compromise between primary and tertiary ones in terms of sorbent regeneration [20].

In this work, a systematic study on the amine modification of GO cryogels is presented to enhance the understanding on the effect of functional groups in amine-modified graphene cryogels on their CO_2_ capture performance, namely residual oxygen functionalities and nitrogen bonding configuration. In general, the literature reports GO covalent modification with amines in harsh conditions of temperature or by complicated processes. Due to this aspect, a more simple approach is highly desired. In this work, the amidation reaction was achieved by simple carbodiimide chemistry as non-toxic alternative and aqueous solvent medium was considered in order to avoid auxiliary procedures, high costs and generation of large chemical wastes. To this purpose, GO was subjected to a carboxylation process and further modified with varying amines, namely ethylenediamine (EDA), diethylenetriamine (DETA), and triethylentetraamine (TETA) in order to introduce varying nitrogen content and configuration into the cryogels. A variation of GO with increased oxidation degree was tested in order to confirm the results on the effect of residual oxygen groups. The results show that the CO_2_ capture performance is greatly influenced by the N configuration and residual oxygen functional groups.

## 2. Materials and Methods

### 2.1. Materials

Aqueous slurry of GO nanosheets (3.73 mg mL^−1^) was provided by Graphenea (Donostia, Spain). The improved oxidation degree GO dispersion (1.5 mg mL^−1^) was supplied as well by Graphenea. Further information on the characteristics of the dispersions is available on the provider website. Chloroacetic acid (ClCH_2_COOH, ≥99.7%), hydrocloric acid (HCl, 37%), sodium hydroxide (NaOH, ≥98%), ethylenediamine (EDA, 99%), diethylenetriamine (DETA, 99%), triethylenetetramine (TETA, ≥97%), 1-Ethyl-3-(3-dimethylaminopropyl) carbodiimide (EDC, ≥98%) and N-hydroxysulfosuccinimide (S-NHS, ≥98%) reagent grade were purchased from Sigma Aldrich (Valencia, Spain) and used as received.

### 2.2. Carboxylation of GO

Prior to use, the GO aqueous dispersions (2 mg mL^−1^) were prepared from the aqueous slurry by ultrasonic bath treatment for 1 h. Carboxylated GO nanosheets (named GO-COOH hereafter) were obtained through reaction with chloroacetic acid under strong basic conditions, to convert oxygen containing groups of GO to carboxylic groups [41]. Briefly, 10 g of chloroacetic acid and 12.8 g of NaOH were added in a 100 ml of a 2 mg mL^−1^ GO dispersion and ultrasonicated in a water bath for 3 h at room temperature. The resulting solution was neutralized with HCl and then purified by repeated rinsing with distilled water and filtration. The product obtained was finally dried in an oven at 50 °C. The improved oxidation degree GO was subjected to the same carboxylation process and employed for further use.

### 2.3. Functionalization of GO-COOH with Amines

The schematics of the synthesis of the cryogels can be found in Figure S3 in the Supplementary Materials. For the amine functionalization of GO-COOH by carbodiimide chemistry, the EDC was employed to activate the COOH groups and further form the succinimide ester by reacting with S-NHS. First, 40 mg of GO-COOH was suspended in 20 mL of deionized water and ultrasonicated in a bath for 1 h. Then 40 mg of EDC and 40 mg of S-NHS were added to the above suspension and mixed. Next, the amine modifier was incorporated under magnetic stirring in a 1:5 wt. GO-COOH/modifier ratio and was left overnight with continuous stirring in ice bath. Then suspension was heated in an oven at 85 °C for 4 h and hydrogel was obtained. The same preparation procedure was used to functionalize the GO-COOH with EDA, DETA and TETA amine modifiers. Finally, the hydrogel was rinsed thoroughly with distilled water and ethanol.

### 2.4. Methods

Three-dimensional porous cryogels were obtained from their respective hydrogels by freeze-drying at −80 °C under a high vacuum at 0.05 mbar in a LyoQuest freeze-drier (Telstar, Madrid, Spain) with three-directional cooling with a rate of about 12 degrees min^−1^ followed by sublimation at 20 °C for 48 h at 0.015 mbar.

The apparent density of the cryogels considered their weight and volume. The cryogel volume was measured with a calliper with an accuracy of 0.05 mm (measurement error ±10%.).

The FTIR spectra were acquired on a FT/IR-6200 (Jasco, Madrid, Spain) spectrometer in the spectral window of 4000–400 cm^−1^ in attenuated total reflectance ATR mode, using a resolution of 4 cm^−1^ and 32 scans.

Thermogravimetric analysis (TGA) was performed on a TGA Q50 thermogravimetric analyzer (TA Instruments, Cerdanyola del Valles, Spain). Samples (5−10 mg) were weighed in titanium crucibles and heated under nitrogen atmosphere from 50 to 800 °C at a heating rate of 10 °C min^−1^.

X-ray photoelectron spectroscopy (XPS) measurements were performed on the powder samples using a spectrometer (VG-Microtech Multilab 3000) equipped with a monochromatic Al X-ray source (1486.6 eV) (Thermo Fisher Scientific Inc., Waltham, MA, USA). The calibration for the surface charging of the binding energy was performed with reference to the C1s peak binding energy. Curve deconvolution for atomic composition was performed using CASAXPS 2.3.17 software (Casa Software Ltd. Wilmslow, Cheshire, UK) by applying a Shirley baseline subtraction and a Gaussian–Lorentzian (70%:30%) peak shape.

The nitrogen and CO_2_ adsorption/desorption isotherms were performed on ASAP 2420 analyzer (Micromeritics, Norcross, GA, USA). The samples were outgassed under vacuum at 80 °C for 24 h before adsorption measurement. The Brunauer–Emmett–Teller (BET) approach was employed to obtain the specific surface areas of modified cryogels from the nitrogen adsorption isotherms measured at 77 K. The CO_2_ adsorption isotherms of the modified cryogels were recorded up to 1 bar at varying operating temperatures.

## 3. Results and Discussion

### 3.1. Synthesis and Characterization of GO-COOH

**Figure 1:**
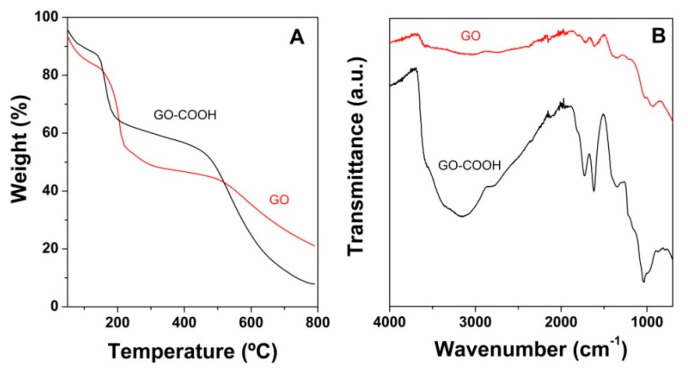
Thermogravimetric curves (**A**) and FTIR spectra (**B**) for graphene oxide (GO) and GO-COOH.

A carboxylation procedure was applied to the parent GO nanomaterial. This approach was applied to normalize the content of oxygen in the GO subjected to amine modification. Moreover, this process was indicated to improve aqueous dispersion stability with respect to GO [42,43], which is highly desirable for the solvo-thermal synthesis of 3D GO-based aerogel structures. Figure 1A shows the TGA curves recorded for GO-COOH and GO nanomaterials. GO starts its decomposition at about 170 °C and continues up to about 300 °C. A second pronounced weight loss occurs around 500 °C. GO-COOH on the other hand starts its decomposition earlier at about 120 °C and loses around 20% of its mass up to 170 °C. Then, a slight but gradual weight loss appears. Both materials show an initial weight loss associated with some structural water evaporation and thermal decomposition of more labile oxygen-containing functional groups [44,45,46]. In the case of GO-COOH, this decomposition starts at a lower temperature due to the higher density of carboxylic acid functional groups attached to its surface. The slight gradual weight loss above 200 °C is attributed to the degradation of more stable oxygen functionalities. Furthermore, at about 500 °C a significant weight loss is observed [47,48].

Figure 1B shows FTIR spectra of GO and GO-COOH, in both spectra the characteristic peaks associated with graphene oxide can be seen. The peak for C=O stretching in carboxylic acid appears at 1728 cm^−1^ and the C=C stretching in aromatic rings occurs at 1618 cm^−1^. Peaks related with C–O–C from epoxy and C–OH from carboxylic groups at 1032 and 1357 cm^−1^ are also showed. Finally, at 3148 cm^−1^ and 2780 cm^−1^ it can be observed a broad band from the stretching vibrations of O–H and C–H bonds [49,50]. From Figure 1B, we can see how the peaks associated with the carboxyl groups at 1728 and 1357 cm^−1^ show a clear increase in intensity. Furthermore, the peak related to the aromatic domains of GO at 1618 cm^−1^ also increases its relative intensity with respect to the rest. The carboxylation process appears to result in the partial removal of some oxygen groups and most probably their transformation. However, the GO-COOH exhibited good dispersion stability, in agreement with other reports [42,43].

**Figure 2:**
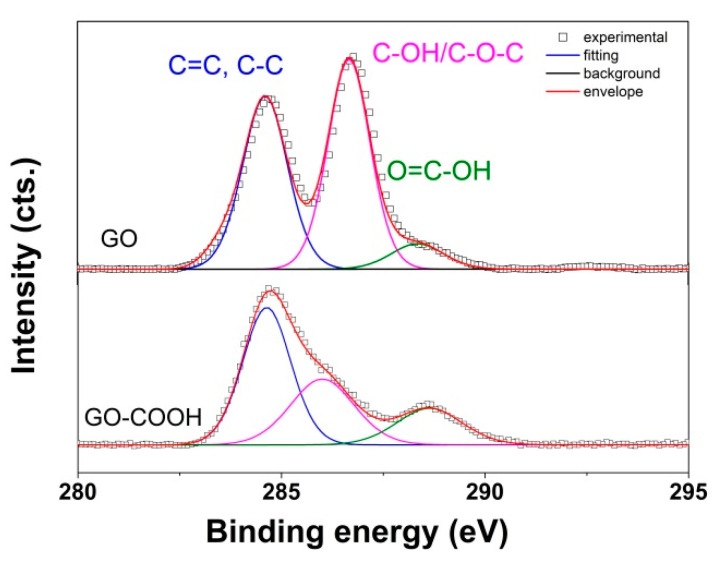
XPS C 1s spectra of GO and GO-COOH.

XPS analysis was further employed to determine the species modified by the carboxylation process. The evolution of the survey and C 1s spectra of GO with carboxylation are presented in Figure 2. The fitting curves employed for the deconvolution of the C 1s peak have a binding energy located at about 284.6, 286 and 288.6 eV and are assigned to C=C/C–C, C–OH/C–O–C and O=C–O, respectively [51]. As can be observed from the corresponding contributions of the deconvoluted C 1s peak, the carboxylic carbon contribution increased upon carboxylation of GO from 7.5 at% to 13.1 at% while some oxygen groups got lost, which resulted in about a 23% increase in C/O ratio, from 2.1 to 2.6 for the GO and GO-COOH, respectively. Thus, the conversion of oxygen groups into others is suggested.

### 3.2. Formation of Amine-Modified GO-COOH Cryogels

**Figure 3:**
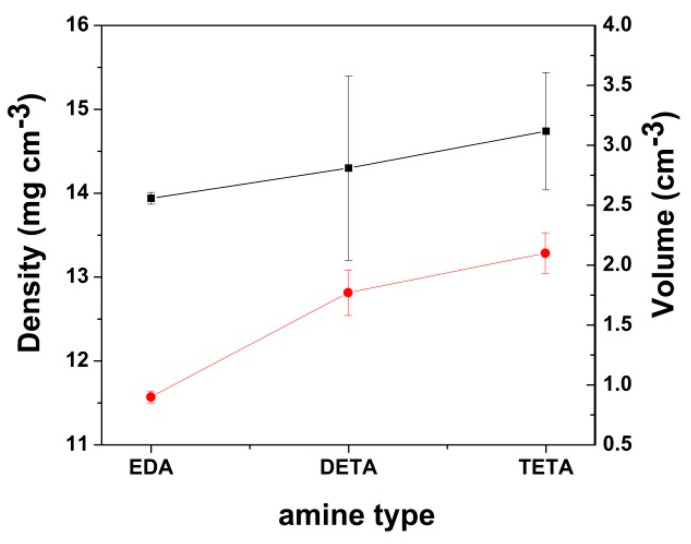
Modified cryogel volume and density with the amine functionalities.

The effect of functionalization with amines of the GO-COOH was further studied on the volume and density of the modified cryogels and it is depicted in Figure 3. It can be seen that both the volume and the density increase with the molecular mass of the amine incorporated in the cryogel, showing the trend EDA < DETA < TETA for the two properties studied. Digital images of the obtained cryogels are available in Figure S4 in the Supplementary Materials.

To explain the obtained trend, two effects associated to the amines must be taken into account. On the one hand, there is the thermal and chemical reduction induced by the amine [14,52] that leads to GO-COOH stacking. On the other hand, there is the effect of the amine molecules introduced between the GO-COOH sheets, which prevent their stacking by acting like spacers [53,54,55]. In this sense, EDA offers the best reducing capacity and, at the same time, is the smallest molecule with the lowest weight.

### 3.3. Characterization of Amine-Modified GO-COOH Cryogels

**Figure 4:**
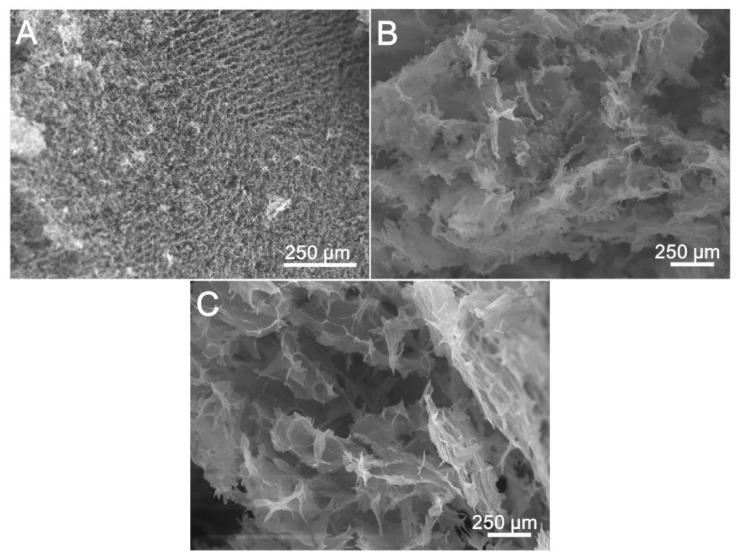
SEM images of GO-COOH cryogels modified with: (**A**) ethylenediamine (EDA) (**B**) diethylenetriamine (DETA) and (**C**) triethylenetetramine (TETA).

The analysis of the effect of the molecular structure of the amines on the morphology of the modified cryogels is further depicted in Figure 4. The SEM measurements indicated that the homogeneity of the distributed sheets decreased, while their stacking increased with the amine functionalities of the modifier, which suggest an improved porosity in the EDA-modified GO-COOH cryogels, while the DETA and TETA-modified ones show enlarged, irregular pores.

**Figure 5:**
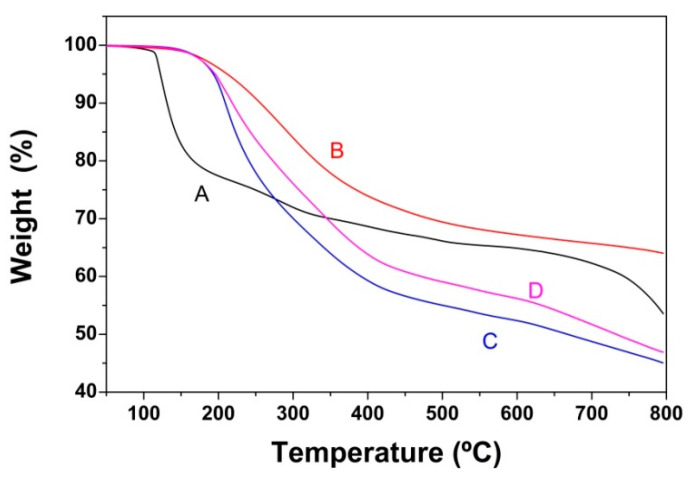
Thermogravimetric curves of GO-COOH before (**A**) and after functionalization with EDA (**B**), DETA (**C**) and TETA (**D**).

Figure 5 shows the TGA curves of amine modified GO-COOH-based cryogels. It can be seen that the amine-functionalized GO-COOH displays a similar profile to that of pristine GO-COOH, while also displaying an improved thermal stability. The amine-functionalized cryogels show a shift towards higher temperatures in the main weight loss compared to pristine GO-COOH, starting decomposition at about 200 °C and going up to 400 °C. However, above 400 °C, only EDA-modified cryogel exhibits enhanced thermal stability, while DETA and TETA-modified GO-COOH show less stability than pristine GO-COOH. The weight loss around 200 °C can be attributed to the degradation of the less stable oxygenated functional groups, as well as to the amine bond [41,42]. Above this temperature, the weight loss is very small and it can be associated with the more stable functional oxygen group decomposition [47,48]. The cryogel functionalized with EDA presents a more improved stability than the rest, indicating a greater number of molecules linked by covalent bonding to the carboxyl groups, forming amide bonds [56]. It was shown that EDA acts as a more efficient reducer for the oxygen functionalities of GO-COOH [49,50]. DETA- and TETA-modified cryogels show a similar and lower thermal stability.

**Figure 6:**
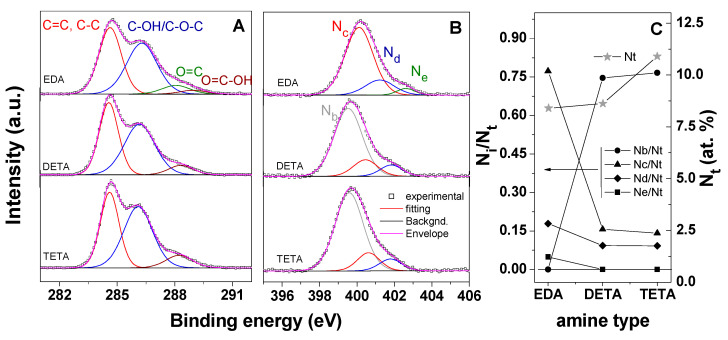
XPS C 1s spectra (**A**), N 1s (**B**) and N configuration contribution (N_i_/N_t_) and total N content (N_t_, at%) for modified cryogels with amine type (**C**).

The XPS analysis of the modified GO-COOH-based cryogels confirmed the successful functionalization with the amines. Figure 6 depicts the C 1s and N 1s spectra for the GO-COOH upon modification with EDA, DETA and TETA, respectively. The results indicated higher atomic C content and lower O atomic content of the cryogels, thus resulting in increased C/O ratio, reaching the values 3.6, 3.8 and 5.1 for the cryogel modified with EDA, DETA and TETA, respectively. This could be explained by the partial reduction in GO-COOH upon modification with the amines [15] and the increased functionalization degree with the modifier, as the amine functional groups increase. The deconvolution of the C 1s peak presented in Figure 6A indicated that the carbon atoms are present in the form of aromatic rings, with the binding energy of the peak located at 284.6 eV, C–OH/C–O–C compared to the binding energy located at about 286 eV, which is also attributed to the C–N peak due to functionalization, C=O with a binding energy at 287.8 eV and O=C–O with a binding energy located at about 288.6 eV [51,57]. The carboxyl content in the amine-modified cryogels decreased with respect to the GO-COOH material, which is attributed to the functionalization with amines. The EDA-modified cryogel appears to exhibit a peak assigned to C=O which is absent in the other cryogels.

On the other hand, the degree of N-doping and nitrogen configuration were studied by XPS, as depicted in Figure 6B,C. The results showed that both total nitrogen content and configurations were strongly dependent on the modifier’s molecular structure. The total N content increased with molecular structure of the modifier, in agreement with the increase in amine functionalities. The N content evaluated by EDAX measurements showed a similar trend, namely it decreased in the order of EDA < DETA < TETA, namely 21.7 < 23.1 < 28.1 (spectra available in the Supplementary Materials).

Moreover, the C/(N+O) ratio increased to 2.5 < 2.7 < 2.9 for the GO-COOH cryogels modified with EDA<DETA<TETA, respectively. It is suggested that, simultaneously with the aqueous functionalization with the amines, the functionalities such as epoxides, hydroxyls and carbonyls on GO-COOH transform into carboxylic acids, which further suffer decarboxylation; thus, a partial reduction takes place in the applied temperature conditions [58].

The N 1s XPS peak was deconvoluted in varying bonding configurations, as shown in Figure 6B,C depicting the evolution of N configuration and their contributions with amine type. The deconvolution of N 1s peak generally exhibits 5 peaks, namely N_a_ or pyridine-N (398.5 eV), N_b_ or nitrile (399.5 eV), N_c_ or pyrrolic–N (400.6 eV), N_d_ or graphitic–N (401.5 eV) and N_e_ or pyrridinic oxide–N (403 eV) [59,60,61,62,63,64,65,66].

The modification with EDA was observed to result in a dominant contribution from pyrrolic-N followed by the graphitic one, while the DETA and TETA-modified GO-COOH cryogels exhibited much lower contributions from such configurations. Except for the nitrile contribution, the other ones in the N configuration decreased in the order of pyrrolic–N > graphitic–N > N–oxide and in the order EDA > DETA > TETA in the modified GO-COOH cryogels. The lower pyrrolic and graphitic N contributions are most probably induced by the occurrence of N–H bonds, which are expected given the structure of the corresponding amines that have increased amine functionalities with respect to EDA [67,68].

### 3.4. CO_2_ Adsorption Properties of Amine Modified GO-COOH Cryogels

**Figure 7:**
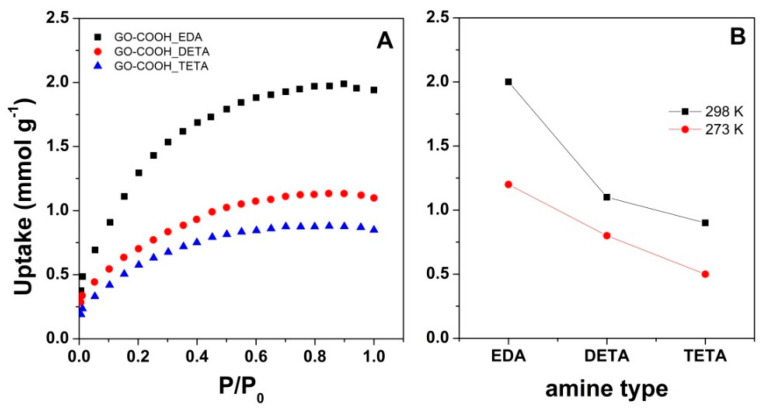
The CO_2_ adsorption isotherm at 298 K (**A**) and evolution of CO_2_ uptake with temperature (**B**) for the amine-modified cryogels.

The cryogels modified with amines by aqueous carbodiimide chemistry were further employed for CO_2_ capture measurements. For exemplification, the effect of the molecular structure of the modifier on the CO_2_ uptake at 298 K is depicted in Figure 7A. As can be observed, the modified cryogel performs the best when its molecular structure contains less amine functionalities, that is, in the order of EDA > DETA > TETA, as in other reports [69]. Moreover, as Figure 7B shows, the modified cryogels exhibited an increase in the CO_2_ capture properties with operating temperature, from 273 K to 298 K. The increase in CO_2_ adsorption with the increase in operating temperature from 273 K to 298 K, irrespective of the amine type, could be attributed to enhanced gas molecule mobility, improved pore filling and the activation of active sites in the amine-modified cryogels with the temperature [9].

The CO_2_ uptake evolution with the amine functionalities could be attributed to various factors. On one hand, the homogeneity properties of the cryogel obtained a lower amine group content as a consequence of the improved dispersion and interaction with oxygen functional groups decorating the GO-COOH sheets, as indicated by SEM results. Many reports linked the active surface area to the improved the adsorption performance [70,71,72]. The surface area obtained from the nitrogen adsorption isotherms at 77 K by the BET theory are presented in Table 1. As can be seen, the surface area increased with the increase in amine functionalities. One may note that the apparent BET surface area values of the cryogels are low and they can be attributed to the low temperature degassing that is used in order to avoid a reduction in GO [73]. Therefore, the BET values could not be directly related to the adsorption properties. Instead, the surface area-normalized uptake (calculated from adsorbed amount at 1bar, 298K divided to BET surface) may be employed to describe the extent of CO_2_ uptake [9,74,75]. It is observed that the surface area utilization factor increases with lower amine functional group content in the modifier, reaching an eight-fold increase for EDA compared to TETA.

The N content is known to influence the adsorption properties, not only in terms of configuration type, but also as contribution values to the total N content [76]. In this respect, the pyrrolic or pyridinic-N configurations were identified as the most important in improving the CO_2_ adsorption, based on the increase in the basicity character of the aerogel surface due to their presence [77]. However, there are contradictory theories in this regard, pointing either to pyrrolic–N [78] or to pyridinic-N [65] as the most favorable configuration. Moreover, CO_2_ adsorption was attributed to take place not only by electrostatic interaction (due to pyrrolic–N and pyridinic–N) but also by dispersion interaction (due to graphitic–N) [79]. In our work, it is shown that the introduction of N atoms into the GO-COOH surface in terms of predominant pyrrolic and graphitic–N greatly enhances the CO_2_ adsorption, with the uptake increasing with their contribution, as indicated by XPS results, showing the evolution as pyrrolic–N > graphitic–N > N–oxide with the increase in amine functionalities in the molecular structure of the modifier, namely EDA > DETA > TETA. Moreover, the obtained results show that the CO_2_ capture performance decreased with the increase in the reduction degree of the modified cryogel expressed not only as C/O, but also as the C/(N+O) ratio, suggesting the marked influence not only of N content, but also of the residual oxygen functionalities on improving the adsorption properties, in line with other reports on the effect of the extent of the reduction degree on improving the adsorption properties [28,29].

In order to obtain more insight into the effect of oxygen functionalities in improving the adsorption properties, the oxidation conditions of GO were modified so as to introduce more oxygen functionalities. In this respect, the same graphite (previously expanded) was employed as it enhances the accessibility of the oxidizing agents. Table 2 indicates the C/O ratio decreased upon using an expanded graphite, as the oxygen content improved. Although the –COOH% of the higher oxidation degree GO is similar to the previous GO, there is a higher contribution from C–OH/C–O–C that could be successfully exploited to increase the –COOH content upon carboxylation of the new material. As a matter of fact, the carboxylation of the higher oxidation degree GO introduced a 2-fold –COOH contribution with respect to GO-COOH as well as a higher dispersion in the contributions of C–OH/C–O–C and C=O. However, –COOH contribution diminished by functionalization with EDA. A reduction took place, as previously shown, which resulted in a C/O ratio higher than its EDA-modified GO-COOH counterpart. This result could be attributed to the increased functionalization degree, as well as lability of the other oxygen functionalities that underwent transformation and decarboxylation.

**Figure 8:**
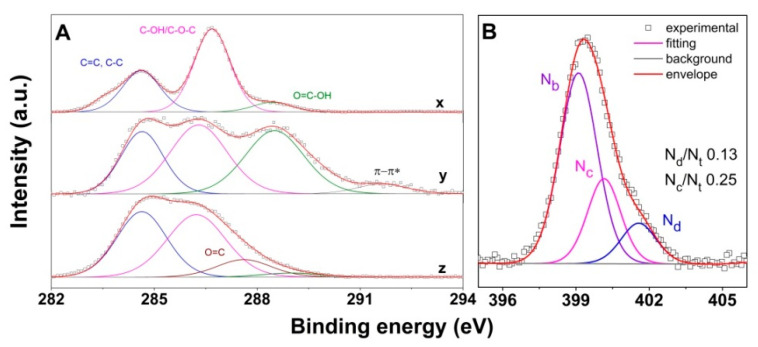
XPS C 1s spectra (**A**) for the higher oxidation degree GO before (x), and after carboxylation (y) and furthermore modification with EDA (z); N 1s spectra upon modification with EDA (**B**).

Figure 8A depicts the evolution of C 1s spectra for the higher oxidation degree GO and its corresponding carboxylated derivative. The deconvoluted C 1s peak shows similar peaks with previous GO-derivatives, namely the C=C/C–C, the C–OH/C–O–C (286.3 eV, which incorporates contribution from C–N induced by functionalization), C=O (287.6 eV), O=C–O (288.5–288.8 eV) and the π-π * shake-up satellite peak (291.5 eV, due to the sp^2^-hybrized C atoms) [58,80,81]. The N 1s spectra of the EDA-modified cryogel obtained from the higher oxidation degree GO is further depicted in Figure 8B. The deconvolution of the N 1s peak indicates the domination of nitriles, as the number of amine groups increased due to enhanced functionalization degree. The occurrence of the nitrile configuration lowers the contributions from pyrrolic and graphitic N to the total N content in comparison to the corresponding counterpart, namely EDA-modified GO-COOH cryogel. The decrease in the pyrrolic and graphitic-N contributions is expected to result in lower CO_2_ capture.

**Figure 9:**
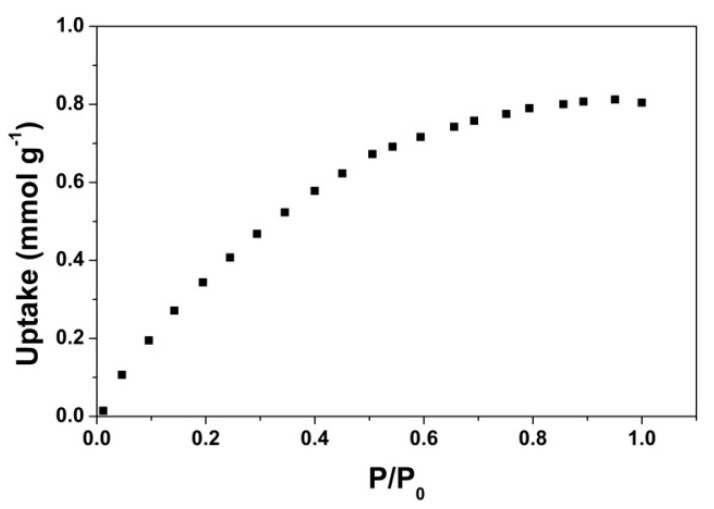
The CO_2_ adsorption isotherm of the cryogel obtained from carboxylation and further modification with EDA of the higher degree GO, at 298 K.

The CO_2_ uptake at 298 K, 1 bar for the EDA-modified new cryogel was obtained as 0.8 mmol g^−1^, as depicted in Figure 9. The performance is lower with respect to the corresponding GO-COOH counterpart. The evolution of the CO_2_ adsorption could be attributed to the lower oxygen functionalities, and decreased contributions from pyrrolic and graphitic-N to the total N content.

## 4. Conclusions

Varying amine functionalization by aqueous carbodiimide chemistry was employed to modify and introduce N content into the structure of carboxylated GO cryogels in order to study the effect of N bonding configuration on the properties and CO_2_ capture performance. The FTIR, TGA and XPS results indicated an increased carboxylic functionality content in the GO-COOH, as the other oxygen groups suffered transformation and partial removal. The functionalization with varying modifiers containing an increased number of amine groups resulted in the further removal of oxygen groups, simultaneous with the introduction of increasing N total content. The XPS analysis revealed a marked influence of the residual oxygen groups and the pyrrolic and graphitic–N bonding configurations of the modified cryogels on their CO_2_ uptake—that is, the best performance was obtained by the cryogel with the lowest C/O and C/(N+O) ratios and highest contribution to total N content from the pyrrolic-N as the dominant configuration followed by graphitic-N, namely the EDA-modified GO-COOH based cryogel. The active surface utilization factor confirmed the decrease in CO_2_ uptake performance in the order of EDA > DETA > TETA. The results obtained in this work show promising alternatives in addressing the task of improving the CO_2_ capture performance of novel amine-modified graphene aerogel.

## Figures and Tables

**Table 1:** BET surface area and surface area utilization factor at 298 K, 1 bar of GO-COOH cryogels with amine modifier type.

Modifier	BET Surface Area, m^2^ g^−1^	Surface Area Utilization Factor, mmol CO_2_ m^−2^
TETA	42.54	0.012
DETA	25.78	0.029
EDA	21.37	0.094

**Table 2:** Evolution of C% and O% atomic composition, –COOH contribution and C/O ratio for higher oxidation degree GO before and upon carboxylation and further EDA modification (XPS based).

Sample	C%	O%	–COOH%	C/O
Higher oxidation degree GO	63.9	34.6	7.6	1.84
Upon carboxylation	65.6	34.4	21.7	1.9
Upon modification with EDA	75.6	17.1	2.1	4.44

## Supplementary Materials

Supplementary data are available online at https://www.mdpi.com/2079-4991/10/8/1446/s1.
