# Supplementary material for: Effect of Varying Amine Functionalities on CO2 Capture of Carboxylated Graphene Oxide-Based Cryogels
Source: Nanomaterials (Basel). 2020 Jul 24;10(8):1446. doi: 10.3390/nano10081446 (PMC7466278; doi:10.3390/nano10081446)
Supplement: Supplementary file 1 [file nanomaterials-10-01446-s001.pdf]

Effect of varying amine functionalities on CO_2 capture of carboxylated graphene oxide-based cryogels

Alina Pruna [1], A. Barjola[1], Alfonso C. Cárcel [1], Beatriz Alonso[2] and Enrique Giménez [1*]

[1] Universitat Politècnica de València (UPV), Instituto de Tecnología de Materiales, Camino de Vera s/n, 46022 Valencia, Spain; apruna@itm.upv.es (A.P.); acarcel@upv.es (A.C.); arbarrui@doctor.upv.es (A.B.R.); enrique.gimenez@mcm.upv.es (E.G.)

[2] Graphenea S.A., Paseo Mikeletegi, 83, 20009, San Sebastián, Spain; b.alonso@graphenea.com

* Correspondent author

Supplementary file

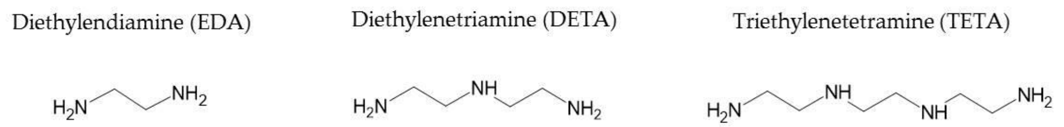

Fig. 1. Structure of the modifiers employed for the functionalization with amine groups

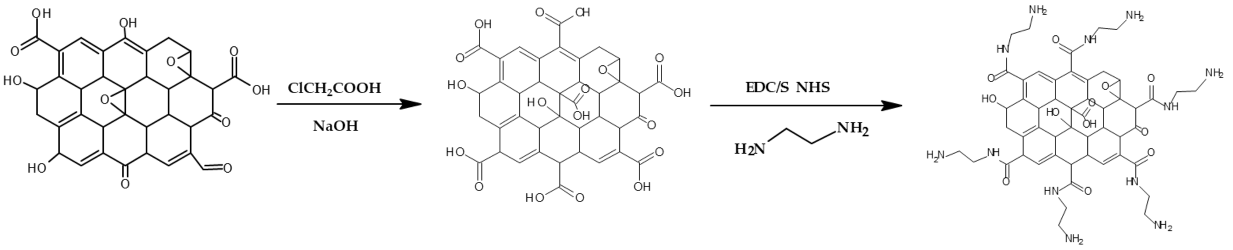

Fig. 2. Scheme for the GO reaction with amine modifiers (e.g. EDA)

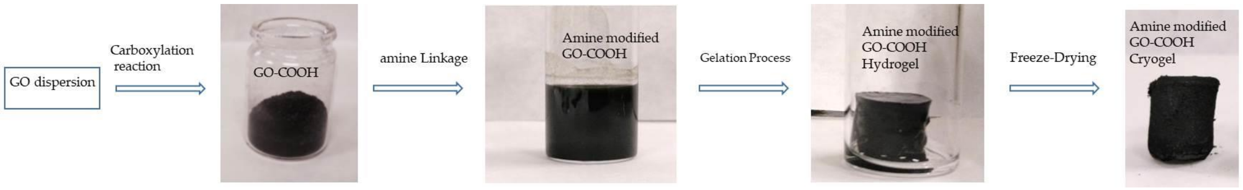

Fig. 3. Schematic of the synthesis process for the amine modified GO-COOH cryogels

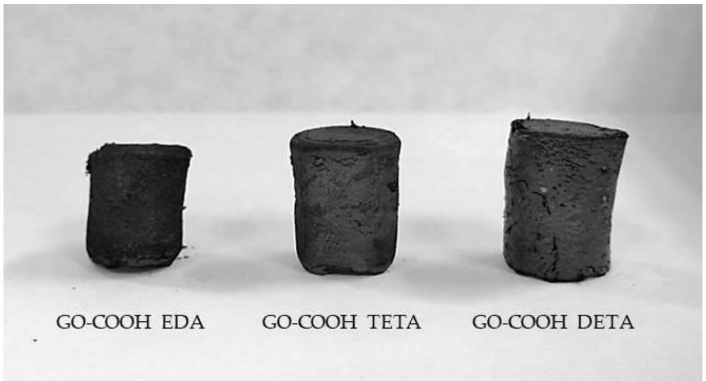

Fig. 4. Digital images of obtained cryogels as a function of amine modifier.

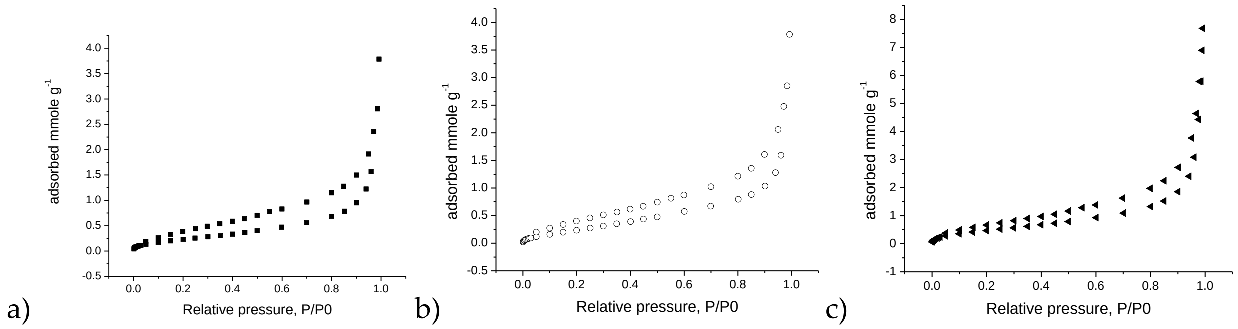

Fig. 5. BET adsorption/desorption isotherms for EDA (a), DETA (b) and TETA (c) modified GO-COOH cryogels.

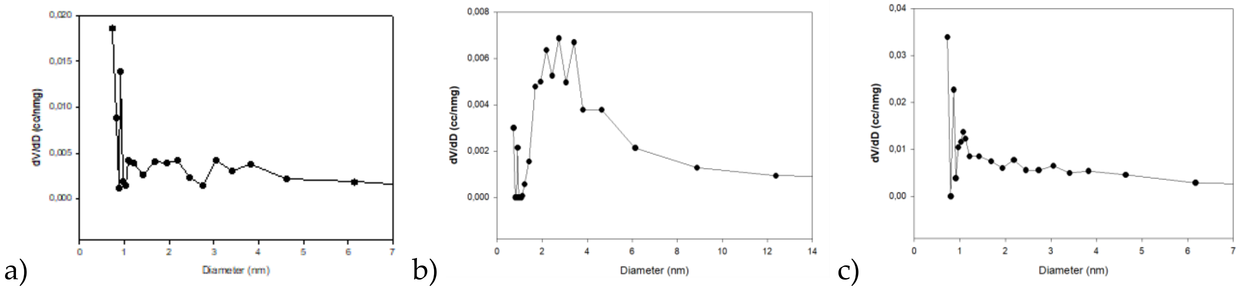

Fig. 6. Pore size distribution from adsorption curves for EDA (a), DETA (b) and TETA (c) modified GO-COOH cryogel.

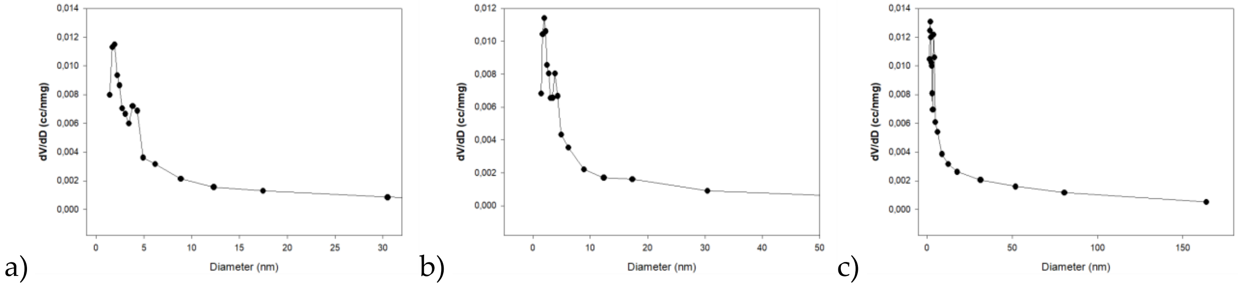

Fig. 7. Pore size distribution from desorption curves for EDA (a), DETA (b) and TETA (c) modified GO-COOH cryogel.

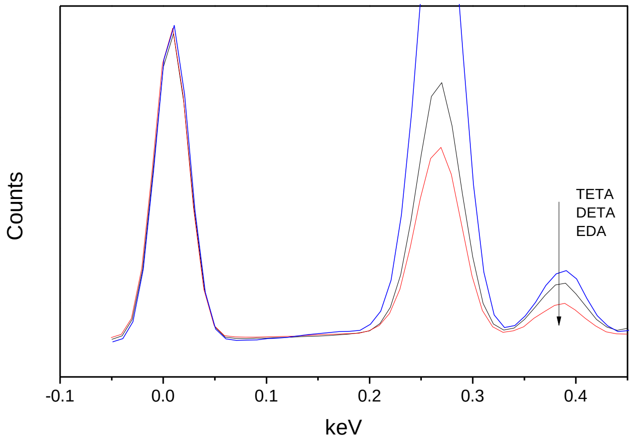

Fig. 8. EDAX spectra showing evolution of N content (arrow indicated) for the GO-COOH cryogel with amine modifier type.
